# IMF-PR: An Improved Morton-Filter-Based Pseudonym-Revocation Scheme in VANETs

**DOI:** 10.3390/s23084066

**Published:** 2023-04-18

**Authors:** Cong Zhao, Jiayu Qi, Tianhan Gao, Xinyang Deng

**Affiliations:** Software College, Northeastern University, Shenyang 110819, China

**Keywords:** VANETs, pseudonym revocation, CRL, improved Morton filter

## Abstract

Vehicle ad hoc networks (VANETs) are special wireless networks which help vehicles to obtain continuous and stable communication. Pseudonym revocation, as a vital security mechanism, is able to protect legal vehicles in VANETs. However, existing pseudonym-revocation schemes suffer from the issues of low certificate revocation list (CRL) generation and update efficiency, along with high CRL storage and transmission costs. In order to solve the above issues, this paper proposes an improved Morton-filter-based pseudonym-revocation scheme for VANETs (IMF-PR). IMF-PR establishes a new distributed CRL management mechanism to maintain a low CRL distribution transmission delay. In addition, IMF-PR improves the Morton filter to optimize the CRL management mechanism so as to improve CRL generation and update efficiency and reduce the CRL storage overhead. Moreover, CRLs in IMF-PR store illegal vehicle information based on an improved Morton filter data structure to improve the compress ratio and the query efficiency. Performance analysis and simulation experiments showed that IMF-PR can effectively reduce storage by increasing the compression gain and reducing transmission delay. In addition, IMF-PR can also greatly improve the lookup and update throughput on CRLs.

## 1. Introduction

The intelligent transportation system (ITS) integrates a variety of advanced equipment and technologies and has gradually become an important part of the next generation of urban transportation, providing convenience for drivers and passengers [1]. Vehicular ad hoc networks (VANETs), as the key parts of ITSs, adopt dedicated short-range communication technology (DSRC) and enable rapid interconnection between vehicle-to-vehicle (V2V) and vehicle-to-roadside infrastructure (V2I) to ensure that drivers and passengers have access to continuous and reliable services and applications [2]. In order to preserve vehicle identity privacy, VANETs mandates that vehicles utilize pseudonyms instead of real identities to anonymize identities and regularly change pseudonyms to prevent tracking from adversaries [3]. However, when vehicles are attacked, an effective pseudonym-revocation scheme should be developed to quickly remove illegal vehicles from VANETs [4]. The following two mechanisms are typically adopted in pseudonym-revocation schemes.

(1) An automatic revocation mechanism [5,6,7]. If an illegal vehicle is identified, the authority only stops issuing new pseudonyms. When the old pseudonym expires, the illegal vehicle will be automatically revoked. An expired automatic revocation mechanism can help the authority to drop the maintenance cost effectively. However, since the legal vehicles are unable to receive the revocation notice timely, the illegal vehicle can continue to misbehave before the old pseudonym expires.
(2) A certificate revocation list (CRL)-based pseudonym revocation mechanism [8]. By distributing CRLs containing illegal vehicles’ identities, the authority is able to remove illegal vehicles from VANETs in time. According to [9], the CRL is typically distributed under the following three conditions.

- Key compromise. The vehicle’s private key or certificate is disclosed or damaged.
- Association change. The information of the vehicle is modified or becomes invalid.
- Service termination. The usage scenario of the certificate or private key is terminated.

However, the traditional CRL-based pseudonym-revocation schemes still face the following issues [10,11,12,13].

(1) Low transmission efficiency. Since all pseudonyms and expiration dates of revoked vehicles are stored in CRLs, it is difficult to rapidly disseminate CRLs to vehicles when driving at high speeds.
(2) Long update period. In order to guarantee communication security, the authority needs to update CRLs regularly. In a CRL-update period, the authority needs to remove the expired illegal vehicles and add new illegal vehicles. According to IEEE Std 1609.2 [8], all illegal vehicles’ information is stored in the entries of CRLs in a linear manner. As a result, when it comes to removing expired pseudonyms, the authority has to go through all the entries. If there are many illegal vehicles in VANETs, the CRL update efficiency will be severely influenced.
(3) Low query efficiency. Before communicating with other surrounding vehicles $v_{j}$, the legal vehicle $v_{i}$ first checks whether the pseudonym and expiration date of $v_{j}$ are recorded in CRLs. Due to the linear storage structure of CRLs, $v_{i}$ needs to traverse all entries. In VANETs, such an inefficient query leads to high authentication and communication delay.
(4) High storage overhead. Since authority must store all records of CRLs, CRLs issued over numerous publishing periods may contain a huge quantity of information about the same illegal vehicle, resulting in the waste of storage. Furthermore, since there is a limited number of vehicles interacting during a CRL life cycle, legal vehicles need to store a great amount of redundant illegal-vehicle information, which causes excessive storage overhead.

In order to solve the above problems, the authors of [14] separated and distributed CRLs to vehicles in the form of data streams to reduce communication costs and CRL distribution delay. However, [14] only focused on CRL distribution efficiency and ignored the optimization of CRL’s data structure. Taking advantage of the low computational cost and high verification efficiency of HMAC, [7,15] replaced the CRL’s linear data structure with a hash table. By regularly updating the hash chain, the authority ensures that legal vehicles can obtain relevant information of illegal vehicles in a timely manner. However, the above two schemes are not able to solve high storage and transmission overhead issues caused by the CRL growth. Ref. [16] adopts a Merkle hash tree (MHT) to optimize CRL data structure. However, the MHT-based cannot support the deletion operation. Moreover, pseudonym validity was verified by obtaining proofs from Roadside Units (RSUs), which imposes huge computational and communication overheads on RSUs. Refs. [17,18] adopted the Bloom filter (BF) to optimize the CRL’s data structure. Likely, the BF-based schemes cannot support the deletion operation as the MHT-based scheme. As a result, the authority must rebuild the CRL before distribution, which leads to low CRL update efficiency. Ref. [19] proposed a pseudonym-revocation scheme based on Cuckoo filter. The scheme supports CRL data-deletion operations with a minimal storage overhead and provides higher data query performance by optimizing the CRL data structure. However, this scheme ignores the maintenance cost of the historical CRLs by authority. As historical CRLs continue to multiply, the storage cost of the authority will increase linearly.

Unlike the Bloom filter (BF), which only supports lookups and insertions but not deletions in its simplest form, the Morton filter (MF) supports lookups, insertions, and deletions. In VANETs, it is necessary to delete expired pseudonyms in CRLs in time to avoid huge overheads caused by CRLs storage and transmission. In addition, MF adopts a compressed block format that permits storing a logically sparse filter compactly in memory. Compared to the stock Cuckoo filter (CF), MFs particularly excel for workloads that use large filters. Due to the large number of vehicles in VANETs, a large number of pseudonyms need to be revoked; therefore, compared with BF and CF, MF is more suitable for VANETs. Moreover, to improve the update efficiency, an improved Morton-filter-based pseudonym-revocation scheme for VANETs (IMF-PR) is proposed in this paper. IMF-PR optimizes the Morton filter to improve the efficiency of CRL generation and query. Meanwhile, in order to reduce the CRL storage overhead and improve CRL distribution efficiency, IMF-PR improves the CRL data structure and implements the distributed CRL management mechanism. To be specific, the main contributions are described as follows.

(1) Based on the previous research [20], IMF-PR establishes a distributed CRL management mechanism, in which authority is responsible for maintaining CRLs, including long-term pseudonyms of illegal vehicles, and the base station maintains CRLs containing multiple illegal vehicles’ temporary pseudonyms that are valid within the scope of base-station management, so as to reduce the computational overhead of authority on CRLs and the communication overhead of distributing CRLs.
(2) IMF-PR improved the Morton filter to optimize the CRL management mechanism. Temporary storage space is used to support the dynamic management of illegal vehicles’ information, so as to keep CRL generation and update efficiency and reduce the CRL storage overhead.
(3) CRLs in IMF-PR store illegal vehicles’ information based on the improved Morton filter’s data structure rather than sequential structure to improve the compression ratio of CRLs and lower the transmission cost of CRLs. Furthermore, the entities owning CRLs can determine whether the surrounding vehicles are legitimate by calculating the fingerprint, which improves the query efficiency.
(4) The compression gain and transmission delay results demonstrate that, compared with C${}^{2}$RL [17] and C${}^{3}$RL [19], CRLs in IMF-PR can be more quickly transmitted to the whole VANETs than them. Moreover, the simulation showed that IMF-PR’s query throughput and update throughput are superior to those of C${}^{2}$RL [17] and C${}^{3}$RL [19] in most cases.

## 2. Preliminaries

### 2.1. VANETs

**Figure 1:**
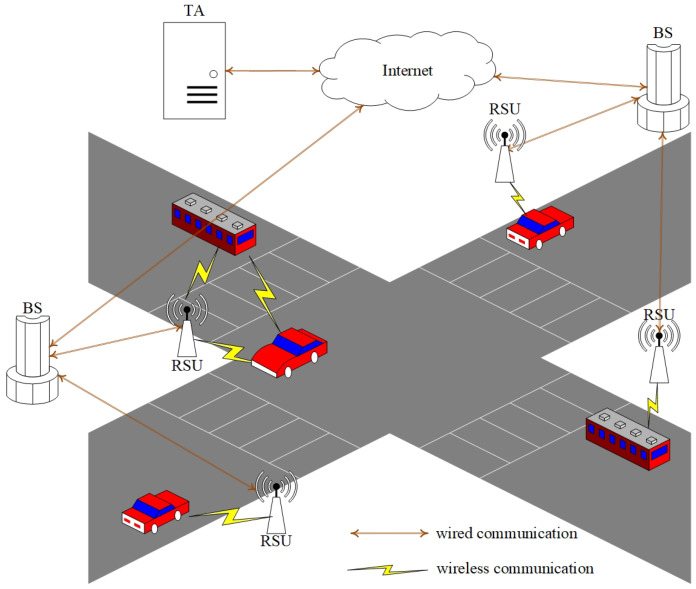
VANET architecture.

VANETs, as essential parts of ITSs, are able to support the connection between vehicles and infrastructures. In VANETs, a broad range of mobile communication technologies are integrated into roadside units (RSUs) and vehicles to relieve traffic congestion and improve driving safety. As shown in Figure 1, vehicles with onboard units (OBUs) adopt dedicated short range communication (DSRC) to communicate with RSUs or other OBUs and obtain required services [8]. RSUs provide safety-related services, efficiency-related services, entertainment-related services, and so forth, for surrounding vehicles. For example, to guarantee traffic efficiency and safety, RSUs provide road-congestion management and collision alerts for surrounding vehicles [21]. A road-congestion application can provide the best routes for vehicles, and communication occurs among the vehicles and from vehicles to RSUs. This application’s goal is to decrease congestion and improve traffic efficiency. In order to prevent malicious vehicles from obtaining services, it is necessary to verify the validity of pseudonyms of vehicles applying for services. The collision alert’s goal is to avoid and decrease the number of accidents. This is an application category sensitive to the delay. To reduce the delay, road conditions use vehicle-to-vehicle communication. Moreover, the pseudonym’s validation must be as fast as possible to avoid the illegal user from broadcasting a bogus message. Therefore, in both examples, it is necessary to design a pseudonym-revocation scheme with a low distribution delay and an efficient query. The base station (BS) can communicate with the external network and provide network communication services for vehicles. In addition, a BS can assist authorities in providing CRL generation and distribution services for vehicles. In VANETs, two different types of communication are used to support applications and services: (1) vehicle-to-infrastructure (V2I) communication. V2I refers to the communication between vehicles and infrastructures (e.g., RSUs). When entering the signal coverage range of an RSU, vehicles are able to adopt DSRC technology to request an RSU to obtain the required services. An RSU has external network communication capabilities through interconnection with the surrounding BS and provides necessary services for surrounding vehicles. (2) Vehicle-to-vehicle (V2V) communication. V2V refers to the communication between vehicles, which is completed by vehicles independently without the participation of RSUs. Depending on V2V communication, vehicles can obtain the driving status of surrounding vehicles in time to ensure driving safety and improve traffic flow.

### 2.2. Morton Filter

**Figure 2:**
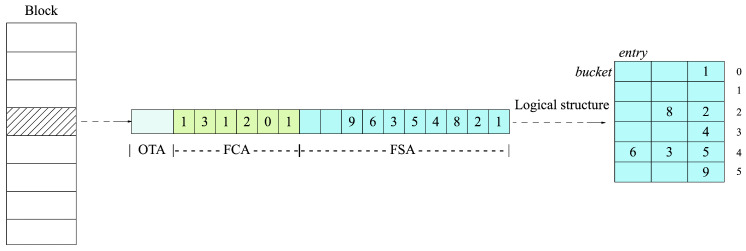
Morton filter.

The Morton filter (MF) [22] is a data structure to support data storage and queries with low spatial complexity and high efficiency. The MF enhances the efficiency of data insertion, querying, and deletion by optimizing a cuckoo filter (CF) [23] and improves space complexity through sparse matrix compression. As shown in Figure 2, the MF takes a block as the basic data unit to store the compressed data. Each block consists of the following three parts.

- Fingerprint storage array (FSA). FSA is made up of a bucket array. Each bucket contains multiple entries, and each entry stores the *n*-bit value (called fingerprint) generated from the hash value of each element.
- Full counter array (FCA). Each slot in FCA is encoded to record the logical structure of the bucket in FSA and tracks the number of fingerprints occupied. FCA facilitates in situ reading and writing of serialized buckets in FSA without materializing the whole logical perspective of related blocks. As a result, FSA does not need to store the empty space in the logical structure.
- Overflow tracking array (OTA). OTA is composed of a bit vector, which tracks the fingerprint overflow status in the block by setting a bit. The fingerprint can be located by querying the value recorded in OTA.

There are three core algorithms in the MF.

(1) Lookup:Given a key *K* and a MF, we first compute the fingerprint $F=H_{F}\left(K\right)$ of *K*. Then, we compute $x=H_{1}\left(K\right)$ to determine the bucket index for its bucket and divide *x* by the buckets per block *B* to yield the block index *b*. Next, we calculate l=mod(x,B) to get the bucket index. We use F,b,l to check for the presence of *K* in the bucket. If not, we recompute $x=H_{2}\left(K\right)$ and then continue with the rest of the operation.
(2) Insert: Given a key *K* and a MF, we first compute the fingerprint $F=H_{F}\left(K\right)$ of *K*. Then, we compute $x=H_{1}\left(K\right)$ to determine the bucket index for its bucket and divide *x* by the buckets per block *B* to yield the block index *b*. Next, we calculate l=mod(x,B) to get the bucket index. We use F,b,l to store *K* in the bucket. If successful, we insert *F*. Else, we recompute $x=H_{2}\left(K\right)$, and then continue with the rest of the operation.
(3) Delete: Similarly to lookup, given a key *K* and a MF, we first compute the fingerprint $F=H_{F}\left(K\right)$ of *K*. Then, we compute $x=H_{1}\left(K\right)$ to determine the bucket index for its bucket and divide *x* by the buckets per block *B* to yield the block index *b*. Next, we calculate l=mod(x,B) to get the bucket index. We use F,b,l to check for the presence of *K* in the bucket. If successful, we delete *F*. Otherwise, we recompute $x=H_{2}\left(K\right)$ and then continue with the rest of the operation.

## 3. System Overview

In this section, the IMF-PR system architecture, the improved Morton filter (IMF) data structure, and the IMF-PR-based CRL (IMF-CRL) are elaborated.

### 3.1. IMF-PR System Architecture

**Figure 3:**
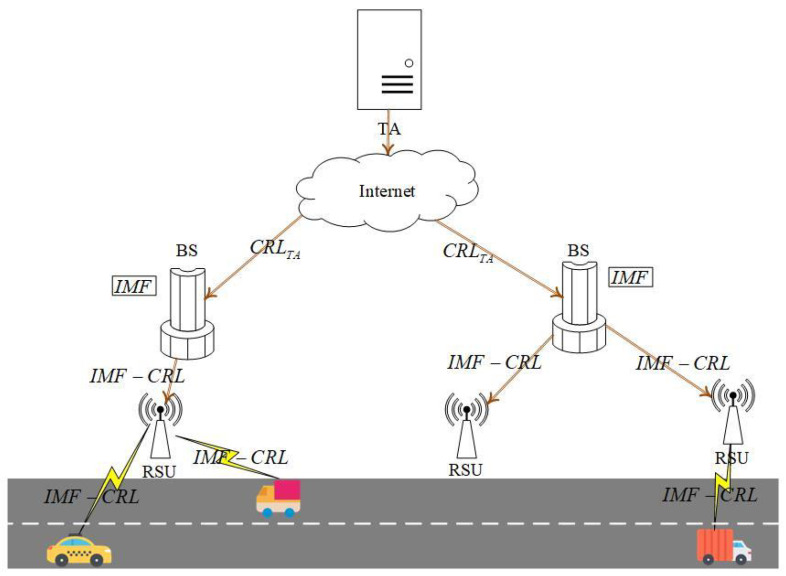
IMF-PR system architecture.

As shown in Figure 3, the IMF-PR system architecture includes four types of entities: TA, BS, RSU, and vehicle.

TA. According to the illegal vehicle’s long-term pseudonym $PS_{\tau }^{TA}$, TA is able to query the real identity of the vehicle and the related long-term pseudonym set ${\left\{PS_{i}^{TA}\right\}}_{i\in [1,n]}$, encapsulate ${\left\{PS_{i}^{TA}\right\}}_{i\in [1,n]}$ and ${\left\{EXP_{i}^{TA}\right\}}_{i\in [1,n]}$ in $CRL_{TA}$, and distribute $CRL_{TA}$ to the BS.

BS. When receiving $CRL_{TA}$ from TA, the BS verifies whether $CRL_{TA}$’s signature is legal. If $CRL_{TA}$ is legal, the BS queries the temporary pseudonym set ${\left\{PS_{i}^{BS}\right\}}_{i\in [1,m]}$ issued by the BS according to the long-term pseudonym ${\left\{PS_{i}^{TA}\right\}}_{i\in [1,n]}$. The BS integrates all long-term pseudonyms, temporary pseudonyms, and expiration dates of all illegal vehicles to generate and distribute IMF−CRL to all RSUs within the managed range.

RSU. When receiving IMF−CRL from the BS, RSU verifies the legitimacy of IMF−CRL’s signature. If IMF−CRL is legal, RSU refuses to communicate with the illegal vehicles contained in IMF−CRL, then distributes the IMF−CRL to all legal vehicles within the communication range;

Vehicle. After receiving IMF−CRL from RSU, the legal vehicle verifies IMF−CRL. Once the verification is successful, the vehicle refuses to communicate with the illegal vehicles recorded in IMF−CRL.

### 3.2. Improved Morton Filter

**Figure 4:**
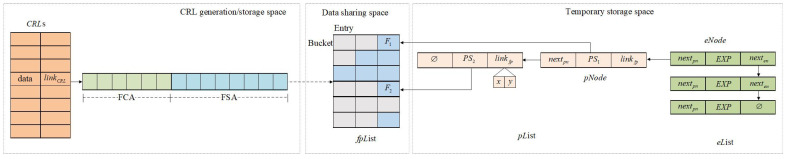
Improved Morton filter.

The improved Morton filter (IMF) data structure that stores the identity information of illegal vehicles in the BS is shown in Figure 4. Three partitions make up the storage space based on the IMF data structure: CRL generation/storage space, data-sharing space, and temporary storage space. If there are new pseudonyms with expiration dates, they are added to the eList and the pList in the temporary storage space. Then, add the corresponding fingerprints to the fpList in the data-sharing space. Finally, update the <FCA,FSA>, and $<date,link_{CRL}>$ in the CRL generation/storage space, which means that the entire IMF-CRL construction process is complete.

(1) CRL generation/storage space stores all history and current IMF−CRL. Differently from the traditional MF, CRL generation/storage space uses a sequential linked list rather than blocks to record all historical and current CRLs. Each item of CRL is composed of two tuples <date and $link_{CRL}>$, where date stores the time when $link_{CRL}$ is created and $link_{CRL}$ saves the address of the sequence lists composed of FCA and FSA. FCA encodes the logical structure of each bucket in fpList to store the number of fingerprints (*F*) in fpList. FSA saves the fingerprints contained in fpList. When the number of entries in a bucket is zero, FSA traverses the entries in the next bucket until all fingerprints stored in fpList are recorded.
(2) The data-sharing space is responsible for dynamically storing the fingerprints of illegal vehicles that need to be revoked in the newest IMF−CRL. Given the pseudonym PS and expiration date EXP of the illegal vehicle, the fingerprint *F* in the fpList (bucket[i]) can be determined by computing $H_{1}\left(PS||EXP\right)$.
  (a) When there are *n* fingerprints in bucket[i], store the fingerprint *F* of pseudonym PS and expiration date EXP in the n+1th slot of bucket[i] in the sequence;
  (b) When there is no remaining slot in bucket[i], execute $H_{2}\left(PS||EXP\right)$ to determine the bucket candidate position, and continue to execute (a) or (c);
  (c) When the bucket corresponding to $H_{2}\left(PS||EXP\right)$ is full, expand the bucket storage space, re-execute $H_{1}\left(PS||EXP\right)$, and store fingerprint *F* in the bucket[i].
(3) Temporary storage space is composed of two linked lists: eList and pList, which are responsible for maintaining the information of the illegal vehicles corresponding to the latest IMF−CRL issued. Each node eNode in eList is composed of three triples: $<next_{pn},EXP$, and $next_{en}>$, where $next_{pn}$ stores the address of the pseudonym linked list pList, EXP is responsible for storing and maintaining the expiration date of illegal vehicles pseudonyms, and $next_{en}$ points to the next eNode in the eList. Each eNode is stored in eList in the order of EXP increment. pNode is composed of three triples:$<next_{pn},PS,$ and $link_{fp}>$, where $next_{pn}$ points to the next pNode with the same EXP, PS is the pseudonym of the illegal vehicle, linkfp stores the address of fingerprint *F* stored in the fpList in the data-sharing space.

### 3.3. IMF-CRL

**Figure 5:**
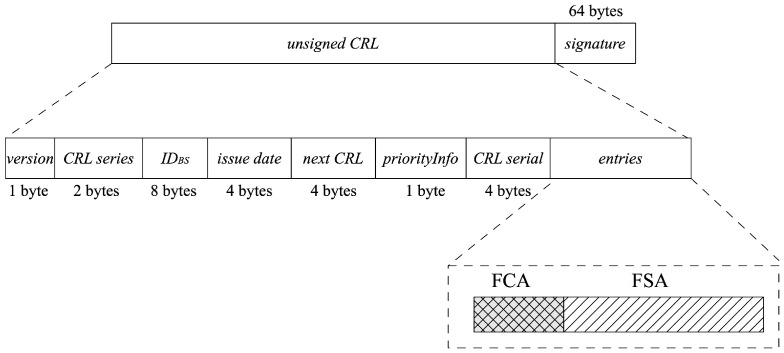
IMF-CRL.

Figure 5 shows the IMF−CRL data structure. IMF−CRL substitutes $ID_{TA}$, identifying the TA’s identity in CRL based on 1609 with $ID_{BS}$ and stores the fingerprints of the illegal vehicles based on IMF data structure instead of the sequence list. The notation and explanations for the IMF−CRL are shown in Table 1.

## 4. The Proposed Scheme

**Figure 6:**
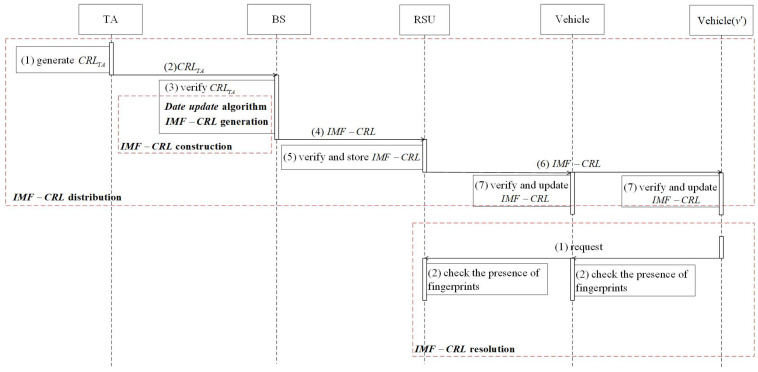
The flow diagram of the proposed scheme.

The flow diagram of the proposed scheme is shown in Figure 6. The proposed scheme includes IMF-CRL construction, IMF-CRL distribution, and IMF-CRL resolution. In addition, the IMF-CRL construction includes a data-update algorithm and IMF-CRL generation.

### 4.1. IMF-CRL Construction

In the CRL generation period, TA follows the WAVE-CRL construction algorithm to load the long-term pseudonyms and expiration date of illegal vehicles into $CRL_{TA}$, and then sends $CRL_{TA}$ to all BSs in VANETs via the secure channel.

When receiving $CRL_{TA}$, the BS first queries the temporary pseudonyms and expiration date issued by the BS in accordance with the entries contained in $CRL_{TA}$. The BS updates eList and pList in the temporary storage space and fpList in the data-sharing space based on the above illegal-vehicle information. Then, given the storage list fpList and current date date, the BS updates CRL generation/storage space and generates IMF−CRL. Finally, the BS generates the IMF−CRL based on the updated fpList.

#### 4.1.1. Data-Update Algorithm

The data-update algorithm consists of the REPF algorithm (remove the expired pseudonym and fingerprint algorithm) and the INPF algorithm (insert a new pseudonym and fingerprint algorithm).

**Figure 7:**
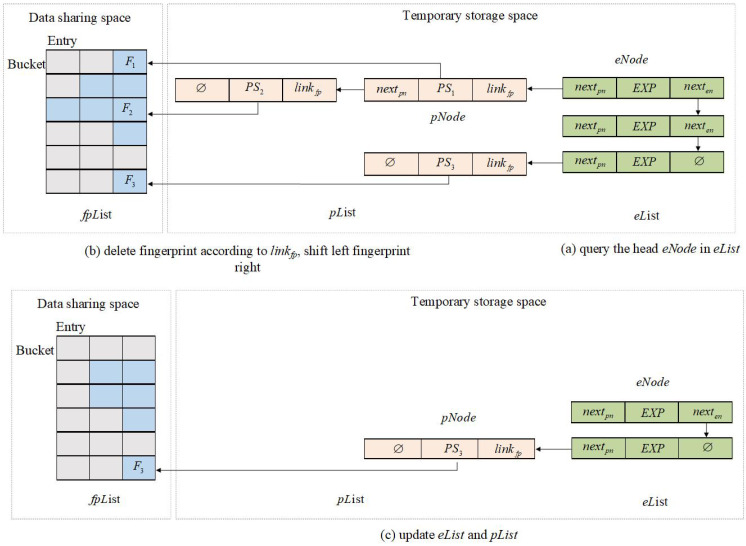
Remove the expired pseudonym and fingerprint algorithm.

Algorithm 1 is in charge of deleting the old pseudonyms, expiration dates, and fingerprints of illegal vehicles stored in IMF. As shown in Figure 7, the details of the REPF algorithm follow.

(1) Query the head node (eNode) in eList. If the EXP stored in eNode is expired, then determine the pList pointed to by $next_{pn}$. For the $link_{fp}$ stored by each pNode in the pList, record the address of the fingerprint *F* in the pNode.
(2) Delete the fingerprint *F* mapped in fpList according to $link_{fp}$, move all fingerprints in the same bucket on the left side of *F* to the right, and set the leftmost slot to null.
(3) Update eList and pList by deleting the head node eNode and all pNode linked to by $eNode.next_{pn}$.

**Algorithm 1** REPF algorithm.**Input:** eList, fpList, pList, currentTime **Output:** eList, fpList, pList 1:eNode←top(eList)2:**if** eNode.EXP≤currentTime **then**3:     **while** $eNode.next_{pn}$ is not empty **do**4:         pNode←top(pList)5:         $bucket\left[x\right]\left[y\right]\leftarrow pNode.link_{fp}$6:         delete fpList[x][y]7:         **if** bucket[x] is not empty **then**8:             bucket[x][y]←bucket[x][y+1]9:         **else**10:           break11:       **end if**12:       pList←popFront(pList)13:   **end while**14:   eList←popFront(eList)15:**else**16:   break17:**end if**18:**return** eList, fpList, pList **end function**

**Figure 8:**
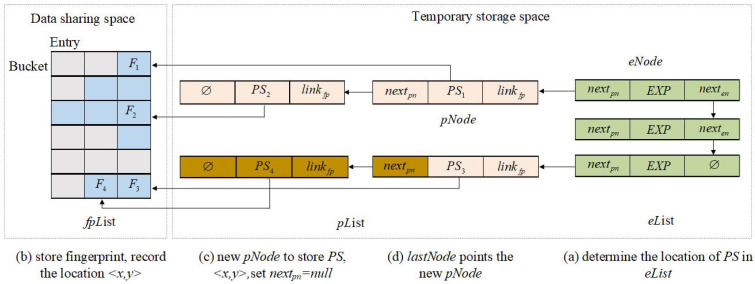
Insert the new pseudonym and fingerprint algorithm.

Algorithm 2 is in charge of storing new illegal vehicles’ pseudonyms, expiration dates, and fingerprints in IMF. As shown in Figure 8, given a new pseudonym PS and expiration date EXP, the BS needs to execute the following operations.

(1) Determine the pList of the new pseudonym PS by traversing the eNode:
  (a) When there exist eNode with EXP in the eList, record the $next_{pn}$ of the last pNode in the pList pointed to by the $next_{pn}$ in eNode as $lastnext_{pn}$;
  (b) When the eList is empty, create a new eNode, add EXP to eNode, set $next_{en}$ and $next_{pn}$ to null, and eList points to the new eNode. Finally, record the $next_{pn}$ in the eNode as $lastnext_{pn}$.
  (c) When there is no eNode with EXP in eList, create a new eNode, add EXP to the eNode, and set $next_{pn}$ to null. Add the eNode in incremental order ($next_{en}$ of the previous node of the eNode points to the node, and the $next_{en}$ of the eNode points to the next node) and record the $next_{pn}$ in the eNode as $lastnext_{pn}$.
(2) Calculate the fingerprints of PS and EXP: $F=H_{F}\left(PS||EXP\right)$ and the address $x=H_{1}\left(PS||EXP\right)$ (or $x=H_{2}\left(PS||EXP\right)$). Store *F* in the first slot of the remaining space (e.g., bucket[x][y]) in the bucket[x].
(3) Create a new pNode, add PS in the pNode, store <x,y> in the $link_{fp}$, and set $next_{pn}$=null (as shown in Algorithm 3 InsertFP algorithm).
(4) The $lastnext_{pn}$ stores the address of the new pNode.

**Algorithm 2** INPF algorithm.**Input:** eList, fpList, pList, PS, EXP **Output:** eList, fpList, pList 1:**if** eList is empty **then**2:    new eNode3:    $eNode.next_{en},eNode.next_{pn}\leftarrow NULL$4:    $eNode\leftarrow (eNode.next_{en},EXP,eNode.next_{pn})$5:    eList←pushFront(eList,eNode)6:    fpList,plist←InsertFP(fpList,plist,PS,EXP)7:**else**8:    **while** traverse eNode in eList **do**9:        **if** eNode.EXP=EXP **then**10:          fpList,plist←InsertFP(fpList,plist,PS,EXP)11:      **else if** eNode.EXP>EXP>nexteNode.EXP **then**12:          new $eNode^{\prime }$13:          $eNode^{\prime }.next_{pn}\leftarrow NULL$14:          $eNode.next_{en}\leftarrow eNode$15:          $eNode^{\prime }.next_{en}\leftarrow nexteNode$16:          $eNode^{\prime }\leftarrow \left(eNode^{\prime }.next_{en},EXP,eNode^{\prime }.next_{pn}\right)$17:          $eList\leftarrow insert(eList,eNode^{\prime })$18:          fpList,plist←InsertFP(fpList,plist,PS,EXP)19:      **end if**20:  **end while**21:**end if**22:**return** eList, fpList, pList **end function**

**Algorithm 3** InsertFP algorithm.**Input:** fpList, plist, PS, EXP **Output:** fpList, plist 1:new pNode2:$pNode.next_{pn}\leftarrow NULL$3:$x_{1}\leftarrow H_{1}\left(PS||EXP\right)$4:**for** y=0;y<entry.length;y++ **do**5:    **if** $bucket\left[x_{1}\right]\left[y\right]\neq NULL$ **then**6:        break7:   **else**8:        $bucket\left[x_{1}\right]\left[y\right]\leftarrow F$9:        $pNode.link_{fp}\leftarrow <x_{1},y>$10:      $pNode\leftarrow (pNode.next_{pn},PS,pNode.link_{fp})$11:      pList←pushBack(pList,pNode)12:      **return** fpList, plist13:  **end if**14:**end for**15:$x_{2}\leftarrow H_{2}(H_{1}\left(PS||EXP)\right)$16:**for** y=0;y<entry.length;y++ **do**17:    **if** $bucket\left[x_{2}\right]\left[y\right]==NULL$ **then**18:        $bucket\left[x_{2}\right]\left[y\right]\leftarrow F$19:        $pNode.link_{fp}\leftarrow <x_{2},y>$20:        $pNode\leftarrow (pNode.next_{pn},PS,pNode.link_{fp})$21:        pList←pushBack(pList,pNode)22:        **return** fpList, plist23:    **else**24:        break25:    **end if**26:**end for** **end function**

#### 4.1.2. IMF-CRL Generation

Given the fingerprint storage list fpList and current date date, the BS generates IMF−CRL as the Morton filter does:

(1) Create a two-tuple <FCA,FSA>, where the size of FCA is the number of bucket in fpList and the size of FSA is equal to the number of fingerprints in fpList;
(2) Traverse fpList and store the fingerprints in FSA in the logical order saved in fplist;
(3) Record the number of fingerprints stored in each bucket in the fpList and store the value in the corresponding slot in FCA;
(4) Package <FCA,FSA> into IMF−CRL.

### 4.2. IMF-CRL Distribution

Based on the WAVE−CRL generation protocol, TA generates and transmits $CRL_{TA}$ to all BSs, which contains the long-term pseudonym set, corresponding to the expiration date set of illegal vehicles, and the signature. After receiving the $CRL_{TA}$, the BS first uses the TA’s public key to verify $CRL_{TA}$. If $CRL_{TA}$ is regarded as legal, the BS queries all temporary pseudonym sets issued by the BS according to the pseudonyms and expiration date stored in $CRL_{TA}$. Then, the BS generates the certificate revocation list IMF−CRL through the IMF-CRL construction algorithm and transmits the IMF−CRL to RSUs. Next, the RSU verifies IMF−CRL. If IMF−CRL is legal, the RSU stores IMF−CRL locally and sends IMF−CRL to surrounding legal vehicles. Finally, the legal vehicle that has received IMF−CRL verifies IMF−CRL. If the verification is successful, the vehicle updates the local IMF−CRL.

### 4.3. IMF-CRL Resolution

When legal vehicle *v* and the RSU receive the request from surrounding vehicles (e.g., $v^{\prime }$), vehicle *v* and the RSU checks whether the fingerprint of vehicle $v^{\prime }$ is recorded in IMF−CRL. Given IMF−CRL, and the pseudonym PS and expiration date EXP of $v^{\prime }$, the process of IMF-CRL resolution is as follows:

(1) Calculate the fingerprint of PS and EXP: $F=H_{F}\left(PS||EXP\right)$;
(2) Compute the index of PS and EXP in FCA: $x=H_{1}\left(PS||EXP\right)$;
(3) By summing the values recorded in FCA[0] to FCA[x−1], the offset of *F* in FSA is derived: $off=\sum_{i=0}^{i<x}FCA[i]$;
(4) Query whether there existing a fingerprint *F* from FSA[off] to FSA[off+FCA[x]−1]:
  (a) If *F* exists, it proves that $v^{\prime }$ is an illegal vehicle; thus, *v* and RSU refuse to communicate with vehicle $v^{\prime }$;
  (b) If there is no fingerprint *F*, calculate $x=H_{2}\left(PS||EXP\right)$, and continue to execute (3) and (4):
    ∗ When the query is successful, it proves that vehicle $v^{\prime }$ is an illegal vehicle. Vehicle *v* and RSU refuse to communicate with vehicle $v^{\prime }$;
    ∗ When the query fails, this proves that the fingerprint of the vehicle $v^{\prime }$ is not recorded in the IMF−CRL; vehicle *v* and RSU execute the V2V and V2I mutual authentication protocols to verify the validity of vehicle $v^{\prime }$.

## 5. Performance Analysis

In this section, IMF-PR, C${}^{2}$RL [17], and C${}^{3}$RL [19] are compared and analyzed in terms of compression gain and transmission delay. Additionally, the simulation framework Veins was adopted to demonstrate the CRL query throughput and CRL-update throughput.

C${}^{2}$RL [17] designed a lightweight online certificate status protocol (TinyOCSP) to save on energy. Then, the C${}^{2}$RL integrates CRL compression using Bloom filters with TinyOCSP to further reduce the certificate validation overhead. C${}^{3}$RL [19] proposed an improved pseudonym certificate revocation scheme, using a Cuckoo filter for compression. In order to optimize deletion efficiency, C${}^{3}$RL [19] designed the concept of the Certificate Expiration List (CEL), which can be implemented with a priority queue. The scheme greatly improves the lookup performance on CRLs and reduces the revocation operation costs through deletion.

### 5.1. Compression Gain

Compression gain ζ is defined as the ratio of the size of WAVE-CRL to the size of the CRL in IMF-PR, C${}^{2}$RL, and C${}^{3}$RL. With the same number of illegal vehicles, the larger the ζ, the lower the space complexity, and the shorter the CRL length.

(1) $$\begin{array}{c} \zeta =\frac{\left|WAVE-CRL\right|}{\left|compressedCRL\right|} \end{array}$$

According to [8], WAVE-CRL consists of three parts: unsigned CRL ($L_{h}$ = 24 bytes), entries comprising pseudonym and expiration date (14 bytes for each entry), and signature ($L_{s}$ = 64 bytes).

In the C${}^{2}$RL scheme based on the Bloom filter, given the array length *m* of the Bloom Filter, the length of C${}^{2}$RL is $l_{h}+\left\lceil \left(m/8\right)\right\rceil +L_{S}$ bytes. Given the number of entries is *n*, the average length of each entry in the Bloom filter is $C_{C^{2}RL}=m/\left(8*n\right)$ bytes. According to [24], $C_{C^{2}RL}$ is defined by the false-positive rate as $C_{C^{2}RL}=m/n=-ln2log_{2}\epsilon$ bits. As a result,

(2) $$\begin{array}{c} \zeta_{C^{2}RL}=\frac{88+14n}{88-\frac{nln2log_{2}\epsilon }{8}} \end{array}$$

In the C${}^{3}$RL scheme based on the Cuckoo filter, assume there are *m* buckets in the Cuckoo filter, each bucket is able to hold up to *b* entries, each entry’s fingerprint length is *f*, and the number of entries is *n*. The length of C${}^{3}$RL is $l_{h}+\left\lceil \left(fmb/8\right)\right\rceil +L_{S}$ bytes; each entry is on average $C_{C^{2}RL}=fmb/\left(8*n\right)$ bytes. According to [25], given a loading factor α, $C_{C^{3}RL}$ is expressed by false-positive rate as $C_{C^{3}RL}=m/n=\left(3-log_{2}\epsilon \right)/\alpha$ bits. Consequently,

(3) $$\begin{array}{c} \zeta_{C^{3}RL}=\frac{88+14n}{88+\frac{(3-log_{2}\epsilon )n}{8\alpha }} \end{array}$$

In IMF-PR, suppose that there are *m* buckets in the IMF, each bucket holds at most *b* entries, the fingerprint length of each entry is *f*, and the number of entries is *n*. The total length of IMF-PR is $l_{h}+\left\lceil \left(f\left\lceil log_{2}b\right\rceil /8\right)\right\rceil +nf+L_{S}$ bytes; each entry is on average $C_{IMF-PR}=\left(mlog_{2}\left(b+1\right)+bf\right)/\left(8*n\right)$ bytes. According to [22], $C_{IMF-PR}$ defines the false-positive rate as $C_{IMF-PR}=\left(log_{2}5-log_{2}\epsilon \right)/4\alpha$ bits. Thus,

(4) $$\begin{array}{c} \zeta_{IMF-PR}=\frac{88+14n}{88+\frac{(log_{2}5-log_{2}\epsilon )n}{32\alpha }} \end{array}$$

**Figure 9:**
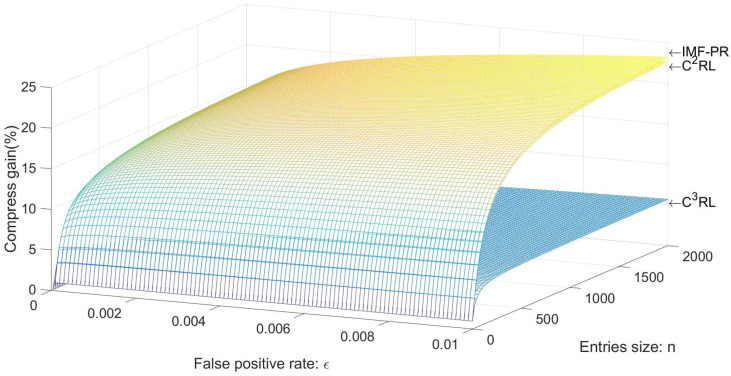
Compression gain (α = 0.5).

The compression gains of IMF-PR, C${}^{2}$RL, and C${}^{3}$RL at the same load factor α = 0.5 are shown in Figure 9. When the number of pseudonyms revoked remains constant, the compression gain rises in tandem with the false-positive rate. As a result, in order to achieve a low false-positive rate, a larger storage capacity needs to be required. Simultaneously, as the false-positive rate increases, the compression gains of C${}^{2}$RL and IMF-PR rise quickly, and the growth rate gradually becomes flat with the false-positive rates of 0.1% to 0.2%. Meanwhile, the overall growth rate of C${}^{3}$RL is modest. With the same false-positive rate, IMF-PR guarantees the maximum compression gain. In addition, as the number of entries stored in the CRL grows, the compression gains of the three schemes starts to increase dramatically and level off when the number of entries reaches 400 to 800. Meanwhile, the compression gain of C${}^{3}$RL increases the slowest and the growth rate of IMF-PR is the highest. When storing the same amount of entries, IMF-PR can provide higher compression gains and shorter the length of the CRL. When the number of entries is 2000 and the false-positive rate is 0.01, the compression gains of C${}^{2}$RL and C${}^{3}$RL are 2.5% and 75.4% lower than those of IMF-PR, respectively.

**Figure 10:**
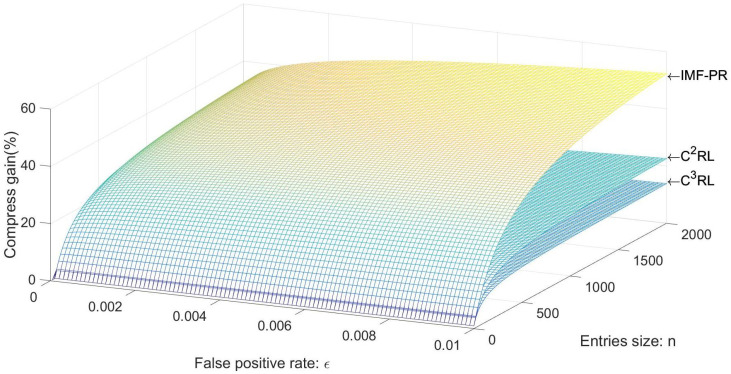
Compression gain (α = 0.8).

Figure 10 shows the compression gains of IMF-PR, C${}^{2}$RL, and C${}^{3}$RL at the same load factor: α = 0.8. Compared with Figure 9, the compression gain of the IMF-PR is significantly increased, and the high compression gain of CRL can be guaranteed while keeping the false-positive rate low. Since the storage space in C${}^{2}$RL has nothing to do with the load factor, the results in Figure 9 and Figure 10 are consistent, whereas the compression gain of C${}^{3}$RL shows a rather smooth increase. In comparison to Figure 9, IMF-PR and C${}^{3}$R achieved higher compression gain with the same storage entry and load factor, while the compression gain of IMF-PR was significantly accelerated and the compression gain of C${}^{3}$RL was relatively slow. When the number of entries was 400 to 800, the compression gain of the three schemes flattened out. When the number of entries was 2000 and the false-positive rate was 0.01, the compression gains of C${}^{2}$RL and C${}^{3}$RL were 56.7% and 73.4% lower than that of IMF-PR, respectively.

### 5.2. Transmission Delay

The transmission delay (TD) is defined as the time from a CRL being distributed to when it is received [26]. In the CRL distribution protocol, the transmission is relatively stable and the delay is small due to the wired transmission among the authority, BSs, and RSUs. This section only discusses the transmission delay from RSU distributing CRL to the vehicle receiving CRL. The TD is defined as

(5) $$\begin{array}{c} TD=\frac{\left|WAVE<CRL>\right|}{bandwidth} \end{array}$$

where |WAVE<CRL>| denotes the data packet size of CRL defined by the WAVE standard. The RSU that receives the CRL needs to layer the data via WSMP, LLC, MAC, and PHY. The RSU then transmits the WAVE-packets to surrounding vehicles via the physical layer. According to the 1069.2 standard [8], given the length of the CRL $L_{CRL}$, the length of the WAVE-packet is $L_{WSA}$=$L_{CRL}+121$ bytes.

**Figure 11:**
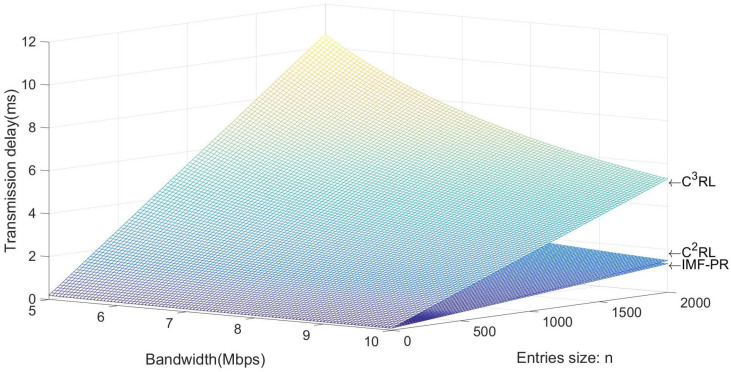
Transmission delay (α = 0.5, ϵ=0.1%).

Figure 11 shows the transmission delays of IMF-PR, C${}^{2}$RL, and C${}^{3}$RL for various bandwidths and entries sizes when α = 0.5, ϵ=0.1%. The transmission delays of the three schemes all show a decreasing trend as bandwidth increases. Since the CRL compression gain of the IMF-PR is the highest when storing the same items, it assures that the VANETs can complete the CRL distribution with the lowest communication cost. Therefore, IMF-PR can complete the distribution of the CRL with lower transmission delay than C${}^{2}$RL and C${}^{3}$RL. When the number of illegal vehicles included in the CRL is 0, the CRLs of the three schemes only contain 121 bytes, so the three schemes can finish the CRL distribution with extremely little transmission delay. As the entries of illegal vehicle stored in the CRL grow, the transmission delays of the three schemes showed an upward trend. C${}^{3}$RL had the most obvious upward trend due to having the lowest compression rate. By owning the highest compression rate, IMF-PR distributes the CRL with the least transmission delay and the slowest rise rate. When the number of entries is 2000 and the bandwidth is 10 Mbps, the transmission delay of IMF-PR is 10.3% and 74.9% lower than those of C${}^{2}$RL and C${}^{3}$RL, respectively.

### 5.3. Simulation

Based on the Veins framework, we conducted simulation experiments on IMF-PR, C${}^{2}$RL, and C${}^{3}$RL in terms of CRL query throughput, where the traffic map of Tianhe District in Guangzhou was embedded as the simulation scenario (2000 × 2000 m${}^{2}$). The simulation tool we used is the open-source framework Vehicles in Network Simulation (Veins) [27]. Veins implements the IEEE 802.11p protocol in the physical and MAC layers and manages the data transmission between OMNET++ and SUMO through TraCI [28]. The data-transmission rate and transmission power are defined as the default values of 6 Mbps and 20 mW, respectively. Then, we define the simulation time and number of cars as 500 s and 20–200, respectively [3,29]. We adopted the open-source MF-library at Github and the main configuration Morton3_8 from the Morton filter proposal paper [22], which means a 3-slot bucket with 8-bit fingerprints. The parameters of the simulation are shown in Table 2.

#### 5.3.1. Query Throughput

Query throughput is defined as the number of CRL query operations completed by RSU and vehicle per unit time [30]. The higher the query throughput and the faster the query speed, the quicker the RSU, and the vehicle can determine whether the surrounding vehicle is legal or not.

**Figure 12:**
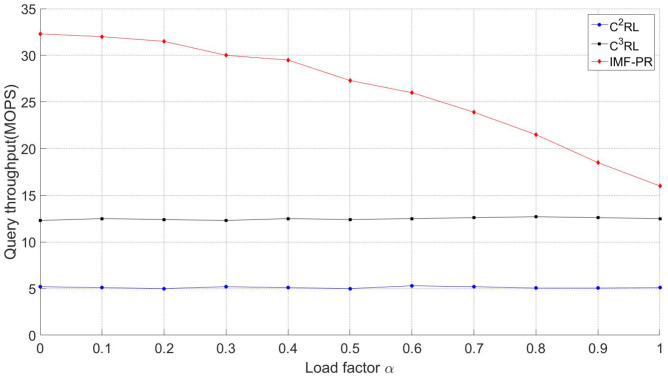
Query throughput.

Figure 12 demonstrates the CRL query throughput of IMF-PR, C${}^{2}$RL, and C${}^{3}$RL under different load factors, α. Both the number of vehicles in the simulation and the running time of the simulation affect the load factor. As the number of vehicles in the simulation and the simulation time increased, the load factor also increased gradually. As the load factor increased, the throughputs of C${}^{2}$RL and C${}^{3}$RL remained relatively stable. However, IMF-PR showed a considerable downward trend, while still maintaining a high query throughput. Furthermore, when the load factor is low, IMF-PR can complete query operations faster than C${}^{2}$RL and C${}^{3}$RL. As can be seen in the figure, when the load factor ranges from zero to one, the query-throughput advantage of IMF-PR over C${}^{3}$RL gradually decreases from 62.5% to 25%, and compared with C${}^{2}$RL, the query-throughput advantage of IMF-PR decreases from 84.4% to 68.8%. Moreover, when the load factor is increased from 0 to 0.5, the load factor of IMF-PR decreases by 10.5%, and when the load factor is increased from 0.5 to 1, the load factor of IMF-PR decreases by 42.8%. As a result, in order to achieve a high query throughput, more storage capacity is required to achieve a lower load factor.

#### 5.3.2. Update Throughput

Update throughput is defined as the number of CRL update operations performed by an authority per unit of time [30]. The higher the update throughput, the faster the authority can update the CRL. The updating of the CRL includes deleting illegal vehicles with expired pseudonyms or falsely reported vehicle information in the old CRL and inserting new illegal-vehicle information. C${}^{2}$RL does not support deletion operations, instead verifying the update throughput by insert operations.

**Figure 13:**
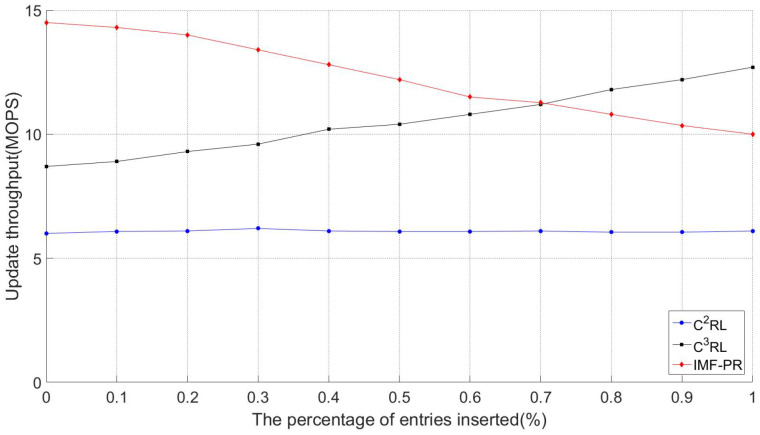
Update throughput. (α = 0.5).

Figure 13 shows the CRL-update throughputs for IMF-PR, C${}^{2}$RL, and C${}^{3}$RL with a load factor of 0.5. Both the number of vehicles in the simulation and the running time of the simulation affect the number of entries that are inserted and deleted. As the number of vehicles in the simulation and the simulation time increase, the number of entries inserted and deleted also increases gradually. However, the percentage of entries inserted does not increase with the number of vehicles and simulation time. It can be seen in the figure that the throughput of C${}^{2}$RL is not affected by the percentage of entries inserted. As the proportion of inserted entries continues to increase, the proportion of deleted entries decreases and the throughput of C${}^{3}$RL continues to increase, which means when the pseudonyms used by vehicles in VANETs have a long validity period—that is, C${}^{3}$RL does not need to delete expired pseudonyms frequently—so the throughput of C${}^{3}$RL is more advantageous. However, in order to protect the privacy of the vehicle, the validity periods of the pseudonyms in VANETs cannot be too long. In IMF-PR, since *eList* in the temporary storage space needs to be updated during inserting entries, the throughput continues to drop. This is because in IMF-PR, entry insertions can only be done one at a time and entry deletions can be done in batches, so throughput decreases as the proportion of entries inserted increases; in contrast, throughput increases as the percentage of entries deleted increases. As can be seen in Figure 13, IMF-PR’s throughput is lower than C${}^{3}$RL’s when the percentage of entries inserted is higher than 70%. When the insertion percentage is zero, the update throughput of IMF-PR is 37.9% and 51.7% higher than those of C${}^{3}$RL and C${}^{2}$RL, respectively. When the insertion percentage is one, the update throughput of C${}^{3}$RL is 20% higher than that of IMF-PR, and the update throughput of IMF-PR is 30% higher than that of C${}^{2}$RL.

## 6. Conclusions

In this paper, an improved Morton-filter-based pseudonym-revocation scheme for VANETs (IMF-PR) was proposed to address the issues of high CRL maintenance, distribution, and query costs. By improving the Morton filter, IMF-PR enhances the efficiency of generating and updating CRL, lowers the CRL’s storage cost, and enables legal vehicles to quickly query and verify the surrounding vehicles. Moreover, IMF-PR supports the decentralized CRL management mechanism, which not only reduces the CRL maintenance cost of TA, but also improves CRL distribution efficiency. The performance analysis and simulation results showed that the compression ratio of IMF-PR is better than those of C${}^{2}$RL and C${}^{3}$RL, and the transmission delay of IMF-PR is 10.3% and 74.9% lower than those of C${}^{2}$RL and C${}^{3}$RL, respectively. In addition, in the worst case, the query throughput of IMF-PR is 25% and 68.8% higher than those of C${}^{3}$RL and C${}^{2}$RL, respectively. Moreover, IMF-PR’s update throughput is better than those of the other two schemes when the percentage of entries inserted is less than 70%. In the future, we will explore a novel anonymous authentication scheme to support secure V2I and V2V communication.

## Figures and Tables

**Table 1:** Notation and explanations for the IMF-CRL.

Notation	Description
version	The version of CRL, which is set to 1 by default.
CRL series	The CRL serial number, indicating whether the CRL is associated with a specific certificate.
$ID_{BS}$	BS’s identity.
issue date	The issuance time of IMF−CRL.
next CRL	Next issuance time of IMF−CRL with the same CRL series.
priorityInfo	Assist legal vehicles with insufficient storage space to identify information that needs to be retained or discarded.
CRL serial	Counter, the value is incremented by 1 when a full IMF−CRL or incremental IMF−CRL is issued.
entries	The vehicles’ fingerprints information.
signature	CRL’s signature.

**Table 2:** Simulation parameters.

Parameter	Values
Hardware platform	CPU: 2.6 GHz Intel(R) Core(TM) i7-6700HQ, 2 GB RAM
Operating system	Debian 9.4
Traffic generator	SUMO
Network simulator	OMNET++
Simulator	veins
Simulation area	2000 × 2000 (m${}^{2}$)
Data Transmission Rate	6 Mbps
Transmission Power	20 mW
Simulation time	500 s
Number of vehicles	20–200

## Data Availability

Not applicable.
